# Lumbar mechanical traction: a biomechanical assessment of change at the lumbar spine

**DOI:** 10.1186/s12891-019-2545-9

**Published:** 2019-04-09

**Authors:** Shigeru Tadano, Hideki Tanabe, Sadao Arai, Keiji Fujino, Tokuhide Doi, Masami Akai

**Affiliations:** 1 Division of Human Mechanical Systems and Design, Faculty of Engineering, Hokkaido University, Kita-13, Nishi -8, Kita-ku, Sapporo-shi, Hokkaido 060-8628 Japan; 2 Tanabe Orthopaedic Clinic, 3-2-16 Narimasu, Itabashi-ku, Tokyo, 175-0094 Japan; 3 Arai Orthopaedic Clinic, 1-19-7 Asumigaoka Midori-ku, Chiba-shi, Chiba, 267-0066 Japan; 4 Fujino Orthopaedic Clinic, 2-15-12 Johoku, Naka-ku, Hamamatsu-shi, Shizuoka 432-8011 Japan; 5 Geriatric Care Facility Excellent Care Shizu, 1316-1 Kami-Shizu, Sakura-shi, Chiba, 285-0846 Japan; 6 Graduate School, International University of Health and Welfare, 4-1-26 Akasaka, Minato-ku, Tokyo, 107-8402 Japan; 7 Present address: National Institute of Technology, Hakodate College, 14-1, Tokura-cho, Hakodate-shi, Hokkaido 042-8501 Japan; 8 Present address: Geriatric Care Facilities Tomisato Tokushu-en, 1-1-1 Hiyoshi-dai, Tomisato-shi, Chiba, 286-0201 Japan

**Keywords:** Chronic low back pain, Lumbar traction, Biomechanical experiment, Finite element method, Traction conditions, Traction stiffness

## Abstract

**Background:**

Lumbar traction is a traditional treatment modality for chronic low back pain (CLBP) in many countries. However, its effectiveness has not been demonstrated in clinical practice because of the following: (1) the lack of in vivo biomechanical confirmation of the mechanism of lumbar traction that occurs at the lumbar spine; (2) the lack of a precise delivery system for traction force and, subsequently, the lack of reproducibility; and (3) few randomized controlled trials proving its effectiveness and utility.

**Methods:**

This study was planned as a preparatory experiment for a randomized clinical trial, and it aimed (1) to examine the biomechanical change at the lumbar area under lumbar traction and confirm its reproducibility and accuracy as a mechanical intervention, and (2) to reconfirm our clinical impression of the immediate effect of lumbar traction. One hundred thirty-three patients with non-specific CLBP were recruited from 28 orthopaedic clinics to undergo a biomechanical experiment and to assess and determine traction conditions for the next clinical trial. We used two types of traction devices, which are commercially available, and incorporated other measuring tools, such as an infrared range-finder and large extension strain gauge. The finite element method was used to analyze the real data of pelvic girdle movement at the lumbar spine level. Self-report assessments with representative two conditions were analyzed according to the qualitative coding method.

**Results:**

Thirty-eight participants provided available biomechanical data. We could not measure directly what happened in the body, but we confirmed that the distraction force lineally correlated with the movement of traction unit at the pelvic girdle. After applying vibration force to preloading, the strain gauge showed proportional vibration of the shifting distance without a phase lag qualitatively. FEM simulation provided at least 3.0-mm shifting distance at the lumbar spine under 100 mm of body traction. Ninety-five participants provided a treatment diary and were classified as no pain, improved, unchanged, and worsened. Approximately 83.2% of participants reported a positive response.

**Conclusion:**

Lumbar traction can provide a distractive force at the lumbar spine, and patients who experience the application of such force show an immediate response after traction.

**Trial registration:**

University Hospital Medical Information Network - Clinical Trial Registration: UMIN-CTR000024329 (October 13, 2016).

## Background

Chronic low back pain (LBP) is a common and major source of distress and disability among working individuals in industrialized countries [1, 2]. In the Japanese population, the prevalence of LBP is highest among other health problems (men: 92.2 per 1000 population; women: 118.2 per 1000 population) according to the statistics by the Ministry of Health, Labour, and Welfare of Japan [3].

Among several conservative treatments for LBP, previous studies have indicated that mechanical lumbar traction is not effective for treating acute LBP [4–6]. A previous systematic review by Wegner et al. concluded that traction, either alone or in combination with other treatments, has little or no impact on pain intensity, functional status, global improvement, and return to work among people with chronic LBP [6]. This finding might be because of (1) the lack of in vivo biomechanical confirmation of the mechanism of lumbar traction that occurs at the lumbar spine; (2) the lack of a precise delivery system for traction force and, subsequently, the lack of reproducibility; and (3) few randomized controlled trials proving the effectiveness and utility of lumbar traction.

However, lumbar traction is still a widely accepted, popular treatment modality for patients with chronic LBP [7, 8]. Many clinicians and physical therapists continue to use it for chronic nonspecific LBP too. Indeed, expert clinical opinions, theoretical models, and research suggest that certain patients with LBP respond positively to lumbar traction [9–11]. Since many patients with LBP are not indicated for surgical intervention, conservative management for patients with LBP is important in clinical practice. Further evidence is needed to support the effectiveness of lumbar traction.

Supposed beneficial effects of lumbar traction are as follows:

- extension of the soft part tissue around the facet joint,
- displaced correction of the intervertebral discs and facet joint,
- separation of the facet joint,
- expansion in the intervertebral foramen,
- decreased pressure of the intervertebral discs,
- extension of the anterior and posterior longitudinal ligaments around the vertebral body,
- reduction of prolapsed discs,
- relaxation of muscle spasm by stretching,
- improvement of blood circulation, and
- psychological effect.

However, some of these in vivo changes are difficult to prove directly.

Recently, we have been able to use a very sophisticated device for lumbar traction with computerized precise control that provides a stable mechanical condition for each individual. One of the main reasons why we could not achieve definite results of lumbar traction previously could be resolved with such a device.

This study aimed (1) to examine the biomechanical change at the lumbar area under such traction and confirm its reproducibility and accuracy as a mechanical intervention; and (2) to reconfirm our clinical impression of the immediate effect of lumbar traction. As a final research object, we plan to investigate the clinical effectiveness of mechanical lumbar traction on pain, dysfunction, and quality of life in individuals with chronic LBP by performing a randomized controlled trial, and the present study is a preliminary step for this.

## Methods

### Study design and setting

This study consisted of two parts. (1) **Biomechanical experiment:** We designed a biomechanical experimental system consisting of a biomechanical assessment of distraction status at the lumbar area in patients with LBP, and finite element modeling at the lumbar spine. (2) **Clinical qualitative study:** We administered a self-report assessment to participants in order to reconfirm the immediate effect of lumbar traction, which is based on our clinical impression, as well as to reconfirm no adverse event.

The current study was part of a comprehensive clinical trial that was registered in the University Hospital Medical Information Network-Clinical Trial Registration (UMIN-CTR 000024329, date opened: October 13, 2016).

### Participants

We recruited participants from six outpatient clinics that used two types of traction devices (MINATO Medical Science, ST-2 L/2CL and OG Wellness Technologies, OL-6500/6000).

### Inclusion and exclusion criteria

The inclusion and exclusion criteria were determined according to a previous clinical trial on chronic LBP [12]. Participants aged 20–65 years who consulted with their orthopedic surgeons because of a complaint of non-specific chronic LBP for more than 3 months’ duration; those who were able to give informed written consent; those without neurologic deficits; and those who satisfied the following criteria: angle of more than 70° during the straight-leg-raising test, negative femoral nerve stretching test result, no superficial sensory deficits, and no muscle weakness less than 4/5 according to the muscle manual testing were enrolled in this study. LBP was defined as pain localized below the L1 spinal process and above the inferior gluteal folds without sciatica (radicular pain). Chronological fluctuation of pain was not considered if the pain itself continued for more than 3 months.

Exclusion criteria were patients who had LBP due to tumors, infections, or fractures; previous back surgery; severe osteoporosis; psychiatric disorders, such as depression or others; liver and renal dysfunction; pregnancy; medication for cardiac failure; a history of cerebrovascular accident and/or myocardial infarction within 6 months before the day of agreement to enter the trial; and were not suitable for traction according to the attending physician.

### Ethical statements

Patients’ attending physicians provided an information leaflet about the experiment, completed the patient information sheets in order to determine eligibility based on the inclusion and exclusion criteria, and obtained written informed consent to participate from each patient. The institutional review board of the Japanese Clinical Orthopaedic Association approved the protocol of the present clinical experiment (approval number: 2015–01).

### Description of the experiment

#### Device for delivering precise traction force

**Fig. 1:**
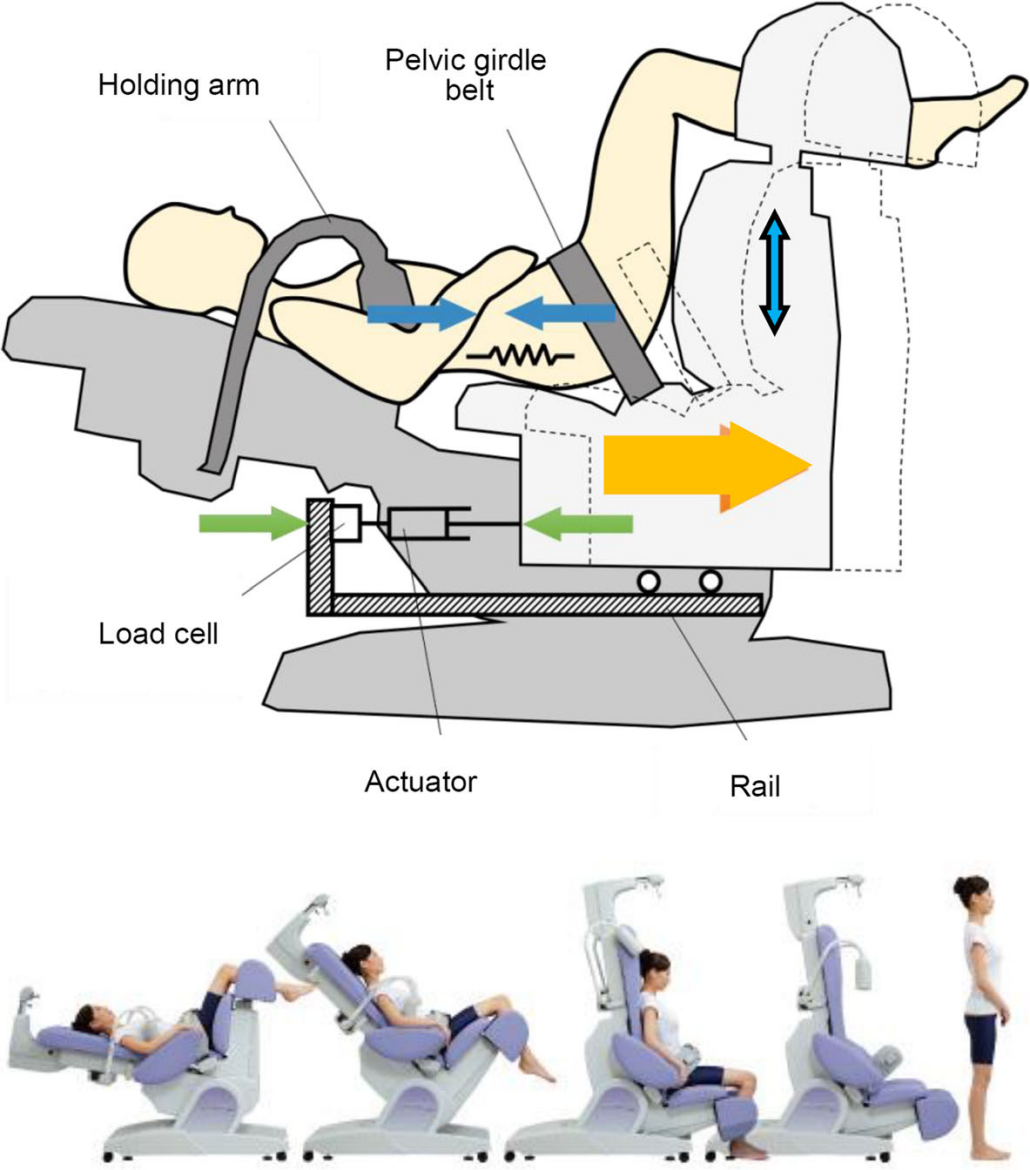
Drawing schema of the traction device used in this study (the bottom of Fig. 1 is adopted from the pamphlet for the ST-2 L/2CL with permission from MINATO Medical Science)**.** The device consists of two main parts: the holding part that holds the upper body and the moving part that maintains a uniform 90°-90° position of the lower extremities. Serial clinical pictures of the application of lumbar traction using the device (ST-2 L/2CL)

We used two types of traction devices that are commercially available as the same category of classification (MINATO Medical Science, ST-2 L/2CL and OG Wellness Technologies, OL-6500/6000). Both device consist of two main parts: the holding part that holds the upper body and the moving part that maintains a uniform 90°-90° position of the lower extremities (Fig. 1). The upper body unit automatically measures the height of the arm pit using an adjustable holding arm to keep the participant in the sitting position. The lower body unit secures the participant in 90° of hip flexion with a pelvic girdle belt, and the thigh lengths with 90° flexion of the knee joints is maintained by lifting the shin component. These two main components are moved separately on the rail via an actuator connected to a load cell.

After the participant laid down on the device, it took 73 s to start the active traction mode. During this phase, the load cell voltage indicated the same pattern of change in each case.

**Fig. 2:**
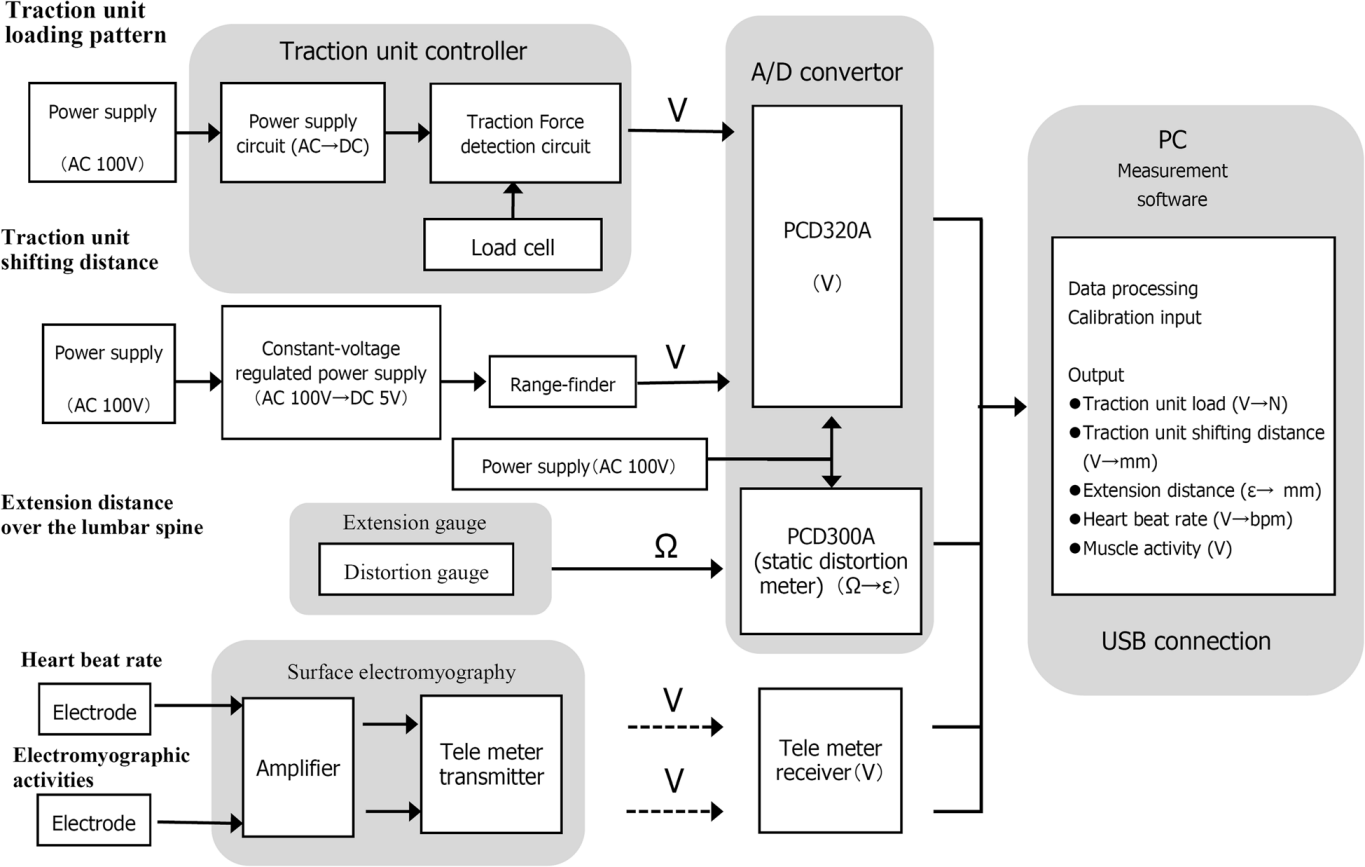
Block diagram of the current measurement system

First, we conducted the biomechanical experiment using the ST-2 L/2CL by incorporating other measuring tools such as an infrared range-finder (SHARP, GP2D12), a strain gauge for large deformation (KYOWA, KFEM-10-120), surface electromyogram (HARADA Electronic, 8-ch EMG Telemeter), electrocardiogram (HARADA Electronic, 8-ch EMG Telemeter), and analog/digital convertor (KYOWA, PCD320A, PCD300A). Figure 2 shows the block diagram of the measuring system. Second, we performed the clinical experiment using the ST-2 L/2CL and OL-6500/6000 in order to calculate in vivo mechanical stiffness and find the difference in traction modes.

The features of the device were as follows.

**Fig. 3:**
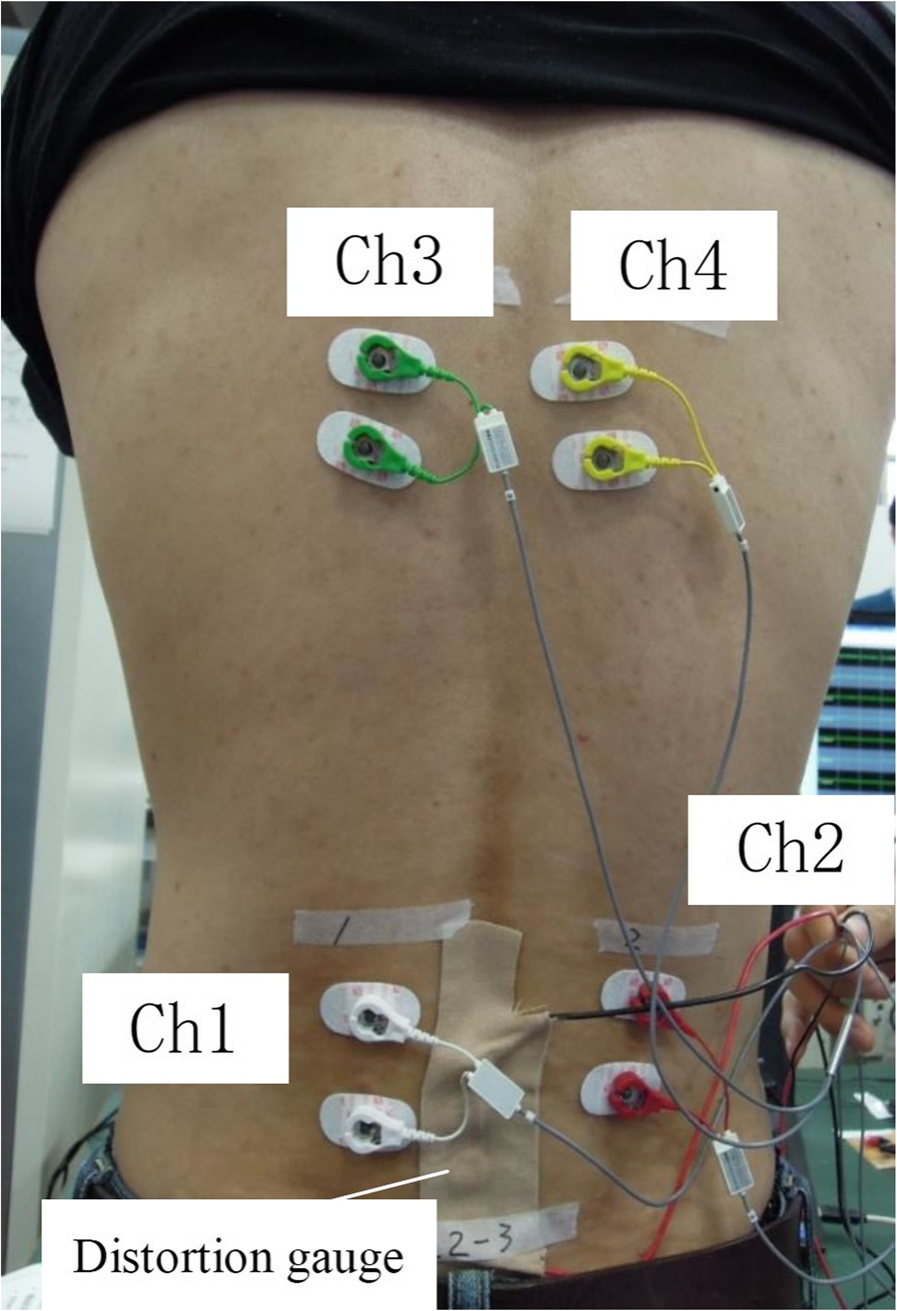
Photograph of a participant’s back. The photograph shows the surface electrodes (Ch. 1–4) for electromyography attached to the paravertebral muscles and the strain gauge attached to the lumbar spine

1. The participant’s posture during traction was maintained in hip flexion of 90° with minimal lumbar lordosis of the moving lower body part. The length of the thigh varied in each case and was adjusted by 90° of knee flexion.
2. The main measuring systems monitored the movement of the traction unit holding the pelvic girdle belt from two aspects: the loading pattern (measured by the machine itself) and distraction distance (measured by the infrared range-finder). The device delivered a regulated traction force between the two main parts via a feedback mechanism against stress-relaxation due to viscoelastic properties of body tissues.
3. Upper body holding at the armpit by the adjustable arm worked to counter the lumbar traction.
  Technicians who had lecture with the staff of Dr. Tadano’s laboratory attended each clinic to add the special devices to the original traction unit. They also supervised the necessary procedures that staff of clinics used to apply the strain gauge system. After exposing the participant’s back, the staff cleaned up the skin with alcohol, pasted the strain gauge with glue, and connected the electrical lines. These procedures were performed in the annex room next to where the traction devices were located.
  The participants in the biomechanical experiment were subjected to the following.
4. A strain gauge was attached at the surface of their lower back with the help of adhesive glue, and it was connected to a static distortion meter (Fig. 3).
5. A surface electromyogram at the three levels of both paravertebral muscles and an electrocardiogram were used to confirm participants’ relaxation status.
  Data generated from these instruments were automatically processed through an analog-to-digital convertor and recorded by a computer as synchronized data.

We measured the following parameters with this system: (1) the traction unit loading pattern, (2) traction unit shifting distance; (3) in vivo extension distance at the skin surface over the lumbar spine, (4) heart beat rate, and (5) surface electromyographic activities.

#### Traction loading pattern

We set two types of loading patterns as a cross-over design, which were applied randomly, with a towing condition determined according to the most popular method of traction used in the present clinical setting (Table 1). We evaluated various experimental conditions as a preliminary study. When we shifted from one towing condition to another one, we allowed at least 20 min of rest as a washout time. In the clinical assessment, at least two days were interposed between the different traction conditions.

**Table 1:** Baseline characteristics of participants of biomechanical experiment and clinical study

	^a^Devices	N	^b^Gender Male: Female	^b^Age (Mean)	Age (SD)	Towing mode traction for 10 min
Biomechanical	ST-2 L/2CL	14	9:5	35.1	11.1	A1, B
		10	4:6	47.7	9.7	A, B
	OL-6500/6000	7	1:6	44.3	11.5	A, B
		7	3:4	40.7	10.4	A, A2
net		38	17:21	41.1	11.7	
Clinical	A → B	49	27:22	42.2	12.6	A, B
	B → A	46	19:27	41.5	13.6	A, B

^a^MINATO Medical Science, ST-2 L/2CL and OG Wellness Technologies, OL-6500/6000

Towing A = Vibration amplitude load was 30% of the preload. It’s repeated for traction 30 s and suspension 5 s

Towing A1 = Vibration function at the frequency of 0.1 Hz was added onto the preload. Vibration amplitude load was 30% of the preload. It’s repeated for traction 60 s and suspension 10 s

Towing A2 = Vibration amplitude load was 20% of the preload. It’s repeated for traction 30 s and suspension 5 s

Towing B = Traction 30 s and suspension 5 s

^b^According to t-test for equality of means between A → B and B → A, age is not significant with mean difference 0.768 (95% Confidence Interval of the Difference: − 4.555 to 6.091), *p* = 0.775

Also, in Chi-Square, there was no significant difference of the male: female ratio between A → B and B → A, *p* = 0.179

For the loads of traction, 40% of the body weight was used as the preload. Ten minutes was used as the traction treatment time.

Towing A1 = Vibration function at a frequency of 0.1 Hz was added to the preload (traction for 10 min). The vibration amplitude load was 30% of the preload (amplitude between 100 and 70%). This traction was repeated for 60 s and suspension for 10 s.

Towing A = The vibration amplitude load was 30% of the preload. This traction was repeated for 30 s and suspension for 5 s.

Towing A2 = The vibration amplitude load was 20% of the preload. This traction was repeated for 30 s and suspension for 5 s.

Towing B = Traction was applied for 30 s and suspension for 5 s.

After setting the various measuring systems, we applied one set of trial traction as the adjustment, and then performed towing conditions A and B three times each for two weeks.

#### Maintaining strain gauge and the measuring conditions

Initially, it was difficult to maintain adhesion of the strain gauge to the skin of the lumbar spine. During distractive application of up to 10 min, some cases failed to maintain the experimental conditions. However, this issue could be controlled for by using tape to reinforce placement of the gauge. Additionally, the electrode wiring clip protruded outside, and there was a possibility that it could come off during towing. During the experiments, the participants wore t-shirts so that the electrode parts did not touch the backrest of the towing machine directly. Occasionally, the electrode clip became pulled off during towing and was unable to obtain a measurement, but in most cases, the myoelectric potential was measured without problems.

The traction delivery chart on display showed some perturbation when the participants strained themselves. Electromyographic activities could be a good indicator for relaxation of the body muscles.

The criteria of successive data recording were dependent on the responsiveness of the infrared range-finder, in which the curve shifted to the + side during the traction phase and to the – side during the relaxation phase.

#### Finite element model simulation

Computer simulation of deformation at the lumbar spine level under lumbar traction force was performed using FEM software (ANSYS 15.0 ANSYS. Inc.). Musculoskeletal modeling of the whole body sitting in the traction device was performed using OpenSim 2.4 (National Center for Simulation in Rehabilitation Research, Stanford University) and CAD software (Geomagic for Solidwork). A whole body skeletal model positioned in the traction device was distorted in the same manner as in clinical treatment.

#### Treatment diary

The participants maintained a treatment diary about their conditions after each intervention for two weeks. This diary was requested to confirm that the device operated without any problems and to collect self-report assessments by participants. They provided brief comments about their health status each day in their treatment diary. Since we added a few parts to the commercial products and modified the traction mode, we mainly investigated the patients’ perception on the newly introduced condition such as the vibration traction method and whether they experienced any inconveniences.

The participants’ perspective on health status before and after lumbar traction, which focused on 6 days of the actual traction trial, was analyzed using a summarized tabulation of their experiences. We used the mixed method with qualitative categorization and quantitative calculation.

An independent researcher read the participants’ comments for each day and classified each one into four categories: no pain, improved, unchanged, and worsened. Coding was used to rate the content of the diary over three consecutive days in order to determine a representative health status: “n” was assigned to no pain, “i” to improved, “u” to unchanged, and “w” to worsened. For example, a figure sequence “uui” meant unchanged – unchanged – improved for three days per week.

### Statistical analysis

Biomechanical parameters (mean values) were analyzed with the paired t-test to determine differences between towing modes A group and B. The 95% confidence interval (CI) of the population mean of the shifting distance, and correlation coefficient between traction stiffness (TS) and age were calculated.

As for the clinical analysis of the treatment diary, the chi-square test was used to determine differences of female-to-male ratio between A → B and B → A. Other data such as age, height, weight, and body mass index were analyzed using the t-test for equality of means between A → B and B → A. The Mantel-Haenszel chi-square for 2 x r tables was used to evaluate the results of the self-report assessments for each traction mode. All statistical analyses were performed with SPSS Statistics 17.0 for Windows (SPSS Inc.).

### Statement of the location where the work was performed

The biomechanical research including FEM simulation was performed in the laboratory of Bio-Mechanical Design, Division of Human Mechanical Systems and Design, Faculty of Engineering, Hokkaido University, and six orthopedic clinics.

The clinical study was conducted in 28 orthopedic clinics (see acknowledgements).

## Results

### Available data acquisition

For the biomechanical experiment, 38 adults aged 20–59 years from six orthopedic clinics participated. The ST-2 L/2CL was used in 24 cases and OL-6500/6000 in 14 cases that provided biomechanical data for calculating stiffness and others. The remaining participants submitted their clinical assessment of the effectiveness of lumbar traction to determine the optimal traction condition (Table 1).

**Fig. 4:**
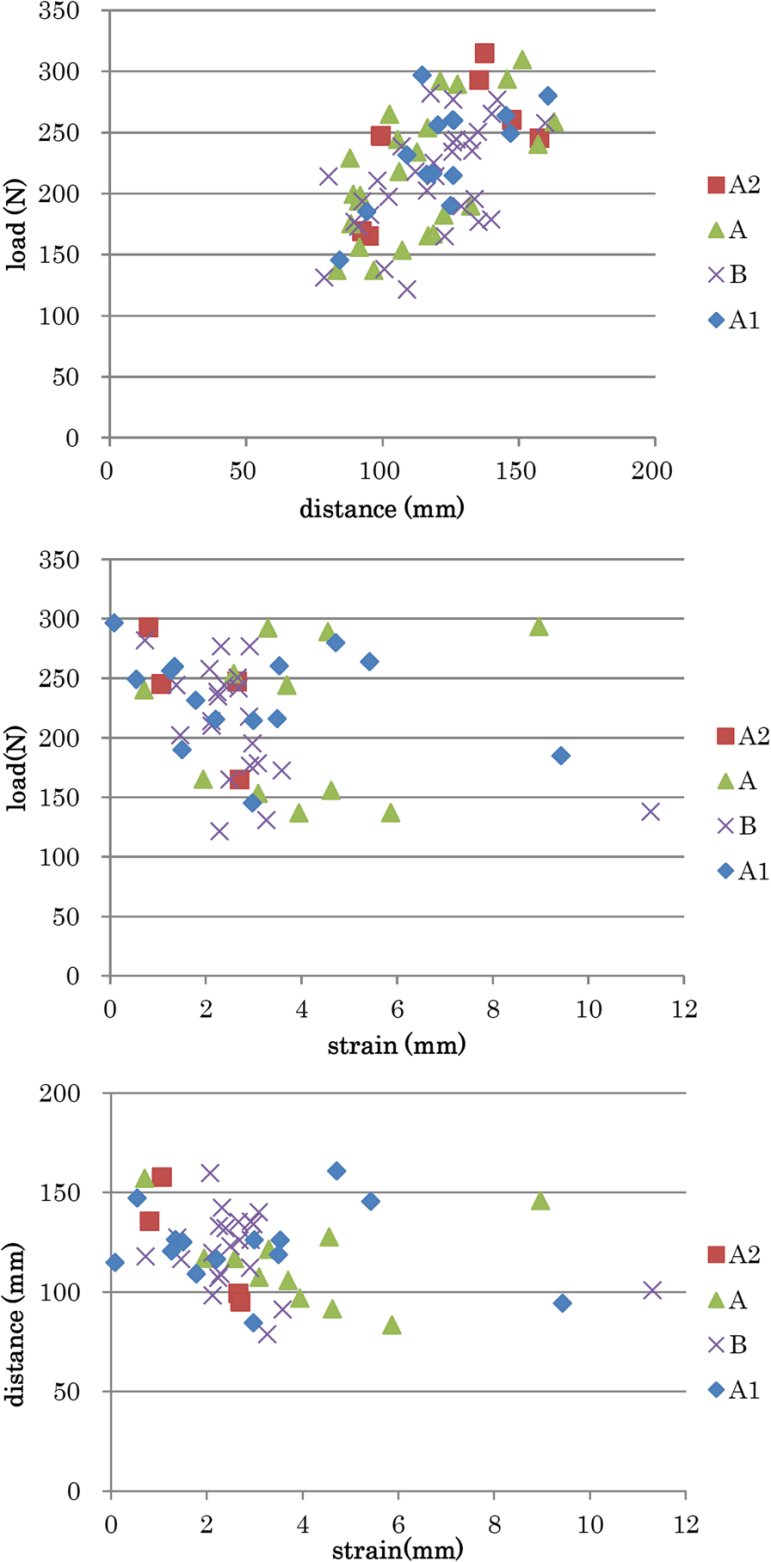
Graphic results of biomechanical measurement. Although there is a proportional relationship between load and distance (i, e., traction stiffness), the relationship between load and strain gauge or between distance and strain gauge is unclear

**Fig. 5:**
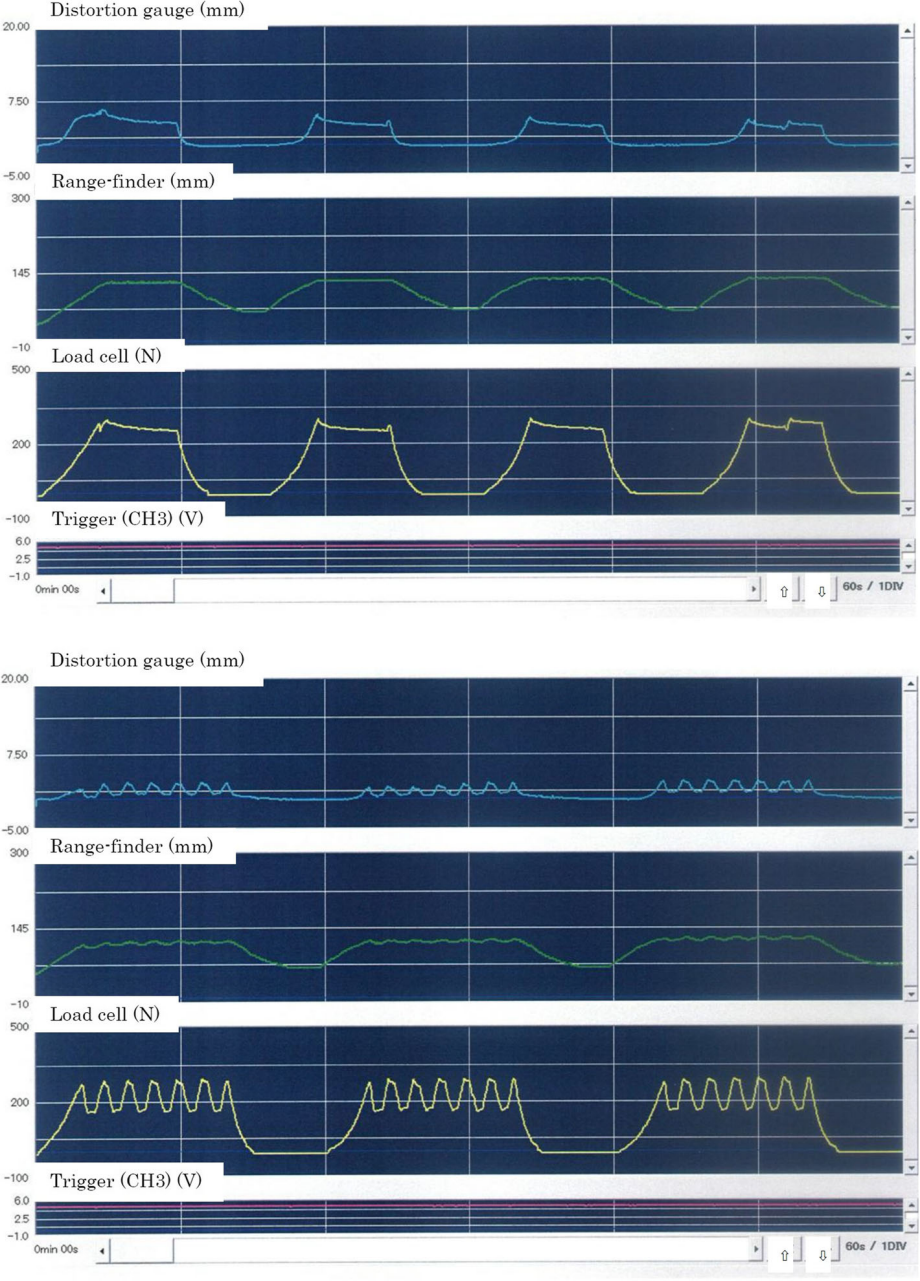
An example of two different towing patterns. The strain gauge was able to determine the linear response to vibration movement at the skin surface of the lumbar spine

Subsequently, we collected biomechanical data from 14 cases for A1 mode, 24 for A, 31 for B, and 7 for A2. The strain data were also available for 14 cases for A1 mode, 8 for A, 22 for B, and 4 for A2 (Figs. 4 and 5).

For the clinical experiment, A to B traction assignment consisted of 49 cases, whereas B to A consisted of 46 cases. We collected 570 health status assessment codes, each after traction condition and converted them into 190 cases with coding of diary content for three consecutive days. Typical comments in the diary were as follows: “I feel so much better,” “The pain was relieved,” “My back got lighter,” “It got easier to move,” and “The numbness became light.” The results are shown in Table 2.

**Table 2:** Self-report assessment of lumbar traction according to qualitative analysis

	Assessment for 3 days			
	Improved	Changeless	Worsened	total
Traction A	78	11	6	95
Traction B	79	12	4	95
total	157	23	10	190

Mantel-Haenszel Chi-Square for 2 x r table. Q_S_ (Mean score statistic) = 0.168, *p* = 0.682

Both A mode and B mode showed improvement in 78 (82.1%) and 79 (83.2%). There was no difference between two modes (*p* = 0.682)

### Results of each parameter

The 95% CIs of the population mean of the distance of the traction unit were 110.9–134.0 mm in A1 mode, 110.1–124.6 mm in B, 103.9–123.5 mm in A, and 98.5–148.8 mm in A2. The 95% CIs of the strain gauge distance were 1.6–4.3 mm in A1 mode, 2.6–6.7 mm in A, and 1.9–3.7 mm in B.

The strain gauge was able to determine the linear response to vibration movement at the skin surface of the lumbar spine during the first step. However, it was difficult to quantify the data of the strain gauge, which may be due to the variations in each subject as well as the viscoelasticity of tissue around the armpit or pelvic girdle at the body holding and other sites that masked the actual fluctuation of living tissue.

### Traction stiffness

TS (N/mm) were calculated using the first peak value of distractive load and shifting distance. In towing A1 with the ST-2 L/2CL, the TS value was 1.96 ± 0.34 N/mm (average value [AV] ± standard deviation [SD]), and it was 2.09 ± 0.11 N/mm in towing A with the OL-6500/6000. In towing B, the TS values were 2.00 ± 0.35 N/mm and 1.94 ± 0.29 N/mm with the ST-2 L/2CL and OL-6500/6000, respectively. There was no statistically significant difference between the two devices (*p*= 0.430 and *p*= 0.656, t-test). There was no relationship between TS and age, as indicated in towing conditions A1 (r = − 0.175), A (r = − 0.300), and B (r = − 0.075).

In the intra-participant comparison, however, the TS value in towing A1 was 1.93 ± 0.09 N/mm (AV ± SD), and it was 1.83 ± 0.08 N/mm in towing B (*p*= 0.001, paired t-test). The TS value in towing A was 2.09 ± 0.11 N/mm, and it was 1.87 ± 0.05 N/mm in towing B (*p*= 0.080, paired t-test). There was no difference between towing conditions A and A2 (*p* = 0.231, paired t-test). In each participant, the difference of traction mode produced distinct responses.

### Finite element method simulation

**Fig. 6:**
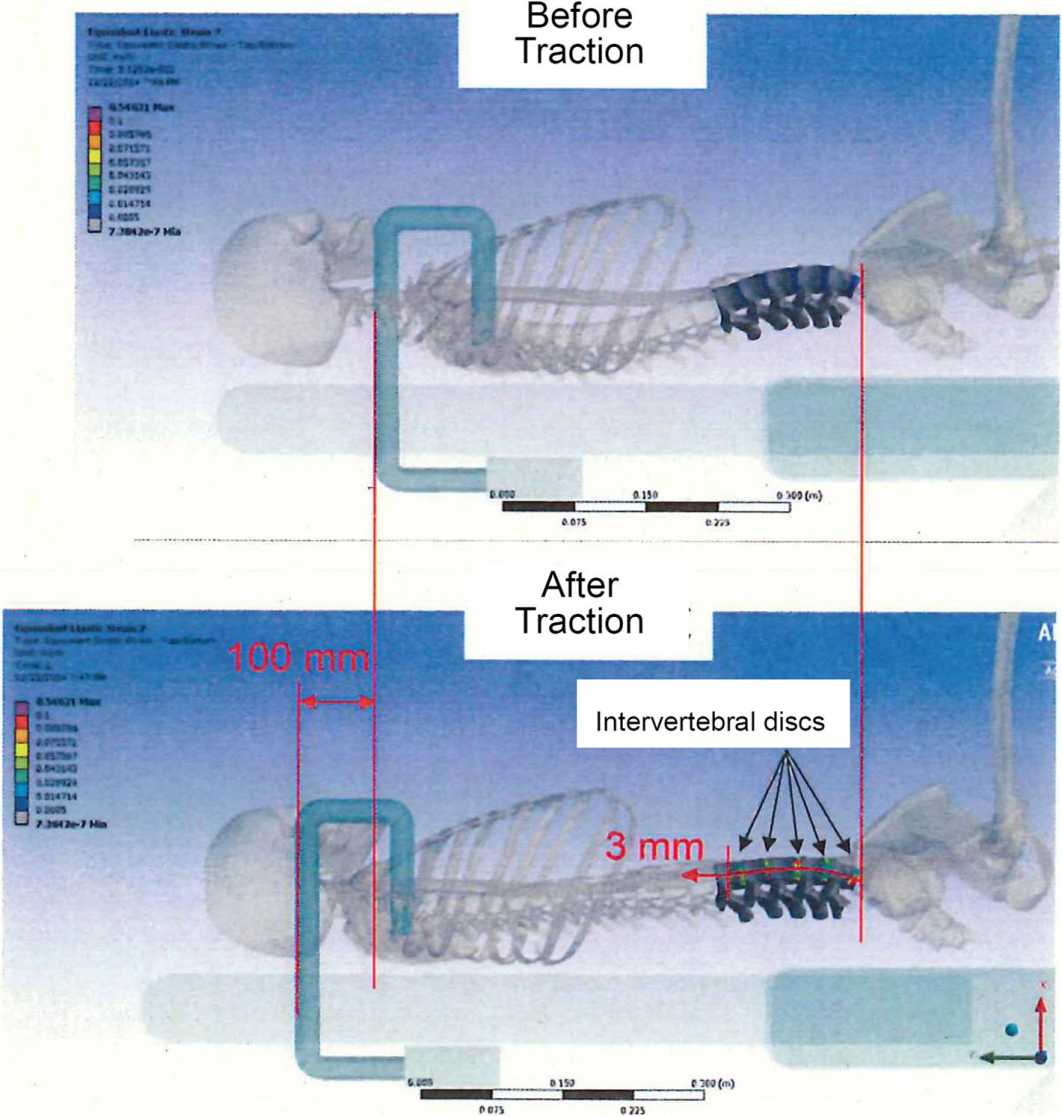
Finite element modeling. Under finite element method simulation, 3-mm displacement of the lumbar spine region is obtained under 100 mm of body traction between the holding arm and pelvic girdle belt. The maximum strain value is 0.55 at the L1/L2 intervertebral disc

In FEM stimulation, when the kyphosis of the entire spine is fixed to the shoulder and the pelvis are under the same condition as the present device, and towing distance is 100 mm, the lumbar region (L1–5) becomes targeted part under distraction. To confirm whether displacement of the spine was driven to a degree, we used the standard whole skeleton shape model (Open Sim Model), which has a physiological curvature (anterior lordosis), as seen in the pre-tow drawing of Fig. 6. In addition, the bone part of the spine had a rigid body that does not become deformed, and the disc had an isotropic elastic body (elastic modulus 500 MPa, Poisson ratio 0.3). We ignored any tissue or intervertebral joint actions such as movement of the ligaments surrounding the spine. All deformation of the spine due to towing occurs in the intervertebral disc. Under these conditions, this analysis confirmed the local deformation (intervertebral disc) against the deformation of the entire spinal column, and the result did not affect the magnitude of the elastic modulus of the intervertebral disc.

According to FEM simulation, 3-mm displacement of the lumbar spine region was obtained under 100 mm of body traction between the holding arm and pelvic girdle belt. The maximum strain value was 0.55 at the L1/L2 intervertebral disc.

### Other findings

Voluntary muscle contraction or movement of the trunk by the participants resulted in turbulence in the traction pattern. Therefore, participants were asked to relax during the experimental period.

### Self-report treatment diary

The coding of diary content emerged in 30 of 64 combinations (=4 × 4 × 4). The frequencies of occurrence of these combination in 190 cases were as follows: nnn, 11; nni, 1; nnu, 3; nin, 4; nii, 4; inn, 7; ini, 1; iin, 10; iii, 30; iiu, 3; iui, 5; iuu, 14; iuw, 1; iwi, 1; iwu, 2; unn, 3; unu, 2; uin, 2; uii, 25; uiu, 9; uiw, 1; uun, 6; uui, 16; uuu, 19; uuw, 2; uww, 1; wnu, 1; wuu, 1; wwu, 3; and www, 2.

The final judgment of each three-day combination was improved with nnn, nni, nnu, nin, nii, inn, ini, iin, iii, iiu, iui, iuu, iwi, unn, unu, uin, uii, uiu, uun, and uui; unchanged with iwu, uuu, wnu, and wuu; and worse with iuw, uiw, uuw, uww, wwu, and www. Both A mode and B mode showed improvement in 78 (82.1%) and 79 (83.2%) cases (Table 2). There was no difference between the two modes (*p* = 0.682). We confirmed immediate utility or change by lumbar traction, which was compatible with the clinicians’ impression in daily practice.

Additionally, there were no cases of stopping and no adverse events.

## Discussion

Despite the long history of use of lumbar traction in clinical practice, the evidence of its effectiveness has not been documented and proved [13]. The Cochrane Database of Systemic Review has indicated that traction may make little or no difference in pain intensity, functional status, and global improvement or return to work when compared to placebo, sham traction, or no treatment [4, 6]. To date, the use of traction as treatment for non-specific LBP is not supported by the best available evidence.

### Interpretation of the current experiment

1. The concept of traction device used in the current study is a link between the upper and lower body segments, which are connected by the lumbar spine. The upper body unit holds the body with holding arms, and the lower body one, which moves on the rail, provides the constant distraction force regulated by the computer against stress relaxation of human tissue. Subsequently, the device is uniformly able to provide machine-delivered traction to the lumbar spine with minimal lordosis. The reasons for the controversial results of the effectiveness of lumbar traction are mainly derived from the lack of availability to maintain constant interventional procedures with a conventional traction device. These potential factors include positioning of the participant, application method of the distraction force, and, sequentially, the lack of confirmation of mechanical loading. With newly developed traction devices, we can more readily measure variables or control these factors.
2. In this study, we qualitatively documented the distraction at the lumbar spine with reproducibility, although the accuracy of the strain gauge on the lumbar skin was not quantitatively sufficient to identify the fine movement of the pelvic traction device. Although distance measurement on the body surface has been considered difficult until now [14], the qualitative aspect on a drawing chart was reasonably supportive for our research.
3. The current experiment was the first stage of our project to examine the effect of lumbar traction. In the next stage, a randomized controlled trial of lumbar traction based on patient-centered outcome and the results of available traction conditions will be needed. The effectiveness of lumbar traction should be evaluated in a combination of a biomechanical study, FEM simulation, and clinical trial.
4. We could obtain a rough figure of shifting distance at the lumbar spine using FEM simulation. However, the validity of the calculated figure should be checked in a future experiment.

### Our current research direction

In this study, we wanted to clarify whether lumbar traction is clinically effective in reducing LBP and determine how lumbar traction affects the body of patients with LBP (mechanism). Given the hesitation to use traction, we first sought to determine the clinical effectiveness even in a subgroup of patients rather than identifying the mechanism of action. After establishing clinical utility, we think it is possible to quantitatively examine the mechanism of action of lumbar traction. According to the system control theory, transfer function provides specific characteristics of the targeted system, which is the lumbar spine in this case. Between input as a loading force and output as a distractive distance, we calculated TS. Even after demonstrating the reproducibility of the traction procedures, we still face the lack of a “good” control (placebo, sham, or null) for lumbar traction that is clinically relevant.

### Bridging the gap to clinical effectiveness

Several previous studies have attempted to assess the biomechanical changes to the lumbar spine, particularly the height of discs, following the lumbar traction procedure [15–17]. Furthermore, researchers have eagerly sought a precise measuring method to detect the changes, including in degenerative models [18–20]. However, even after confirming mechanical loading, there may be variations in the responsiveness of patient groups. Previously, researchers have shown such variations in responsiveness to lumbar traction [10, 11, 21].

By combining the results of a clinical study and FEM simulation, we could confirm some mechanical actions at the lumbar spine in the current study. We have already prepared some measurement scales for clinical evaluations that have already undergone psychometric standardization [4, 12, 22, 23]. If we include the clinical effectiveness of lumbar traction in a well-designed clinical trial, it is possible to have sufficient evidence of the effectiveness of lumbar traction based on the current biomechanical study. Actual loading patterns or the manner in which the lumbar spine experienced loading should be investigated after determining clinical effectiveness.

In this study, we added vibrating force to the preload (corresponding to 40% of the body weight) because some prior articles have reported the usefulness of vibration on LBP, and we think that increasing sensory input could have a positive effect [24, 25].

### Study limitations

Identifying an appropriate loading mode may still be an essential step for ascertaining the clinical utility of lumbar traction. The measurement of distraction distance itself simply relies on a computer simulation conducted by FEM. We only assessed the distance on the lumbar skin and did not directly assess the shift of discs or vertebral bodies. However, the distraction load is applied through the upper and lower body parts that have moved on the horizontal rail. As the position of the lumbar spine maintains minimal lordosis in each case, shifting distance on the skin parallelly reflects positions of bony elements in deeper layers.

## Conclusions

The current study, which combined a biomechanical experiment with FEM simulation and analysis of patients’ perspective, found that lumbar traction operates as an actual mechanical intervention therapy for patients with chronic LBP, and it provided the possibility of an immediate effect after traction.

## Abbreviations

A/D convertor: analog-to-digital converter
AV: average value
CAD: computer-aided design
CI: confidence interval
FEM: finite element model
LBP: low back pain
SD: standard deviation
TS: traction stiffness
